# Geohelminthiasis and Malaria Co-Infection in Pregnant Women in Kinshasa: Case of Lisungi and Lukunga Hospitals in Democratic Republic of the Congo

**DOI:** 10.3390/pathogens15010004

**Published:** 2025-12-20

**Authors:** Clarisse Dianzenza, Japhet Kabalu Tshiongo, Lise Kuseke, Francine Muswele, Gustave Kasereka, Daddy Mangungulu, Eddy Kakiese Laken, Vivi Maketa Tevuzula, Kassoum Kayentao, Hypolite Muhindo Mavoko

**Affiliations:** 1 Department of Tropical Medicine, University of Kinshasa (UNIKIN), Kinshasa P.O. Box 01306, Democratic Republic of the Congo; kabalujaphet@gmail.com (J.K.T.); lisekuseke@gmail.com (L.K.); muswelefrancine@gmail.com (F.M.); gustavekasereka@gmail.com (G.K.); mnglbambi@gmail.com (D.M.); kakieseeddy@gmail.com (E.K.L.); vivi.maketa@unikin.ac.cd (V.M.T.); hypomavoko@gmail.com (H.M.M.); 2 Parasites and Microbes Research and Training Center (PMRTC), University of Sciences of Techniques and Technologies of Bamako (USTTB), Bamako BP 1805, Mali; kayentao@icermali.org

**Keywords:** soil-transmitted helminthiasis, *Plasmodium falciparum*, pregnancy, anemia, Kinshasa, Democratic Republic of the Congo

## Abstract

Background: Geohelminthiasis and malaria are major public health problems in Kinshasa. Pregnant women are particularly vulnerable to these conditions due to their weakened immunity, with severe complications such as maternal anemia and low birth weight. This study assessed the prevalence and associated factors of geohelminth–malaria co-infection. Methods: A cross-sectional study was conducted in two hospitals in Kinshasa, Democratic Republic of the Congo, which included 336 pregnant women. The lab analyses included thick smears for malaria, stool examinations for geohelminths, and hemoglobin measurements. Multivariate logistic regression was used to identify associated variables, with a significance level set at *p* < 0.05. Results: Geohelminth–malaria co-infection was observed in 5.7% of pregnant women, while the prevalence of geohelminthiasis alone was 7.7%. *Ascaris lumbricoides* was the most frequent parasite (6.3%), followed by *Trichuris trichiura* (1.5%) and *Ancylostoma duodenale* (0.3%). The third trimester was associated with a significantly increased risk of co-infection and geohelminthiasis (ORa = 5.8; 95% CI: 1.36–9.23; *p* = 0.0077). No significant association was found between co-infection and maternal anemia. Conclusions: Although co-infection prevalence was low in Kinshasa, the third trimester is a risk period. Systematic screening and integrated management during pregnancy are recommended.

## 1. Introduction

Intestinal parasitic infections, particularly geohelminthiasis, remain a major public health problem in tropical and subtropical regions [1]. The World Health Organization (WHO) monitoring and evaluation framework emphasizes the need for regular surveillance of schistosomiasis and geohelminthiasis control programs, especially in endemic areas [2]. The primary causative agents include *Ascaris lumbricoides*, *Trichuris trichiura*, and *hookworms* (*Ancylostoma duodenale* and *Necator americanus*) [3]. These parasitic infections primarily affect vulnerable populations, particularly pregnant women. Infections with *A. lumbricoides* and *T. trichiura* result from the ingestion of embryonated eggs present in fecally contaminated environments due to poor sanitation. In contrast, hookworm eggs hatch in the soil and release larvae that develop to the infective stage, capable of penetrating intact human skin, typically when individuals walk barefoot on contaminated ground [4,5,6,7]. Despite ongoing control effort, the WHO framework highlights the persistently high prevalence of these parasitic diseases in many low-income countries and calls for strengthened prevention, management, and monitoring among at-risk population, particularly women of childbearing age [2].

Approximately 1.5 billion people are infected with geohelminths, about 24% of the global population. Sub-Saharan Africa (SSA) alone accounts for estimated 553 million cases [4]. Among pregnant women in this region, around 30% are infected during pregnancy [8]. These infections are associated with complications such as anemia, fetal growth restriction, and adverse perinatal outcomes [3,9].

At the same time, malaria remains another major threat to pregnant women in these regions. Physiological changes during pregnancy weaken maternal immunity, increasing susceptibility to malaria and other endemic diseases, including geohelminths [10,11]. In 2023, an estimated 10.6 million pregnant women living in areas of moderate to high transmission in SSA were exposed to malaria, with nearly 39% residing in the Democratic Republic of the Congo (DRC) and Nigeria [12,13].

Co-infection with geohelminths and malaria increases the risk of maternal anemia and worsens obstetric and neonatal prognosis, such as low birth weight (LBW), prematurity, and maternal and neonatal death [10,14]. In the DRC, certain cultural practices, such as geophagy (soil ingestion), frequently observed during pregnancy, expose pregnant women to soil compounds such as tannins. These polyphenolic substances inhibit intestinal iron absorption by forming insoluble complexes, thereby promoting iron deficiency anemia [15]. Simultaneously, this practice increases exposure to parasites present in contaminated soil, further elevating the risk of maternal anemia and obstetric complications [16].

Despite the endemicity of both conditions in Kinshasa, recent and specific data on geohelminth–malaria co-infection among pregnant women are lacking. Moreover, previous studies have rarely examined the interaction between these infections in this local context.

This study aimed to assess the prevalence and associated factors of geohelminth–malaria co-infection among pregnant women in Kinshasa, DRC.

## 2. Methods

### 2.1. Population and Study Design

This cross-sectional study was nested within the PYRAPREG clinical trial (PACTR202011812241529). This multicenter, randomized, open-label phase 3 trial evaluated the efficacy, safety, and tolerability of pyronaridine-artesunate (PA) compared to artemether-lumefantrine or dihydroartemisinin-piperaquine in pregnant women infected with *Plasmodium falciparum* during the second or third trimester of pregnancy (results will be reported separately) [17]. Participants were followed for 63 days from day of inclusion according to a predefined schedule (day 0 to day 63). Malaria detection was performed at each visit, and hemoglobin levels were measured on days 0, 7, 14, 28, 42, 63, and at delivery. Stool samples were collected at baseline (day 0) to detect geohelminths, with a follow-up sample two weeks after treatment for those with positive results.

### 2.2. Study Site

The study was conducted in two health facilities in Kinshasa: Lisungi Medical Center (Mont-Ngafula municipality) and Lukunga Medical Center (Ngaliema municipality) in Kinshasa. Both located in semi-rural suburban areas of the city, these maternity wards provide high-quality healthcare at affordable costs, which explains the high attendance of pregnant women at antenatal care services.

### 2.3. Data and Sample Collection

Recruitment for this study was conducted from 16 November 2022 to 24 February 2024. A total of 336 pregnant women were enrolled, among whom 219 were participating in the PYRAPREG trial and 117 were recruited during the routine antenatal care (ANC), after completion of the trial. Malaria testing is not routinely performed during ANC. At enrollment, blood samples were collected for *Plasmodium* detection, parasite density estimation, and hemoglobin measurement. At the same opportunity, stool samples were also requested from pregnant women. Sociodemographic, obstetric, and behavioral data (including geophagy practices) were also recorded on a case report form.

### 2.4. Laboratory Procedures

#### 2.4.1. Malaria Diagnosis

Thick and thin smears were prepared and stained with 10% Giemsa. Only the thin smear was fixed with methanol prior to staining. Parasite density was estimated from the thick smear for 200 or 500 leukocytes using the formula: Number of trophozoites × 8000)/Number of white blood cells counted [18]. Two microscopists read the slides independently, and a third one played a role in resolving discrepancies (positive or negative results; parasite density > 20%; or species identification).

#### 2.4.2. Hemoglobin Measurement

Hemoglobin level was measured using the Hemocue Hbkit 301 System Kit^®^ (HemoCue AB, Ängelholm, Sweden) [19]. According to the WHO criteria, anemia in pregnancy is defined as hemoglobin level < 11 g/dL: mild (10.0–10.9 g/dL); moderate (7.0–9.9 g/dL) or severe (Hb < 7.0 g/dL) [18].

#### 2.4.3. Parasitological Stool Analysis

Stool samples were collected in 60 mL plastic containers, fixed in 10% formalin solution, labeled with a unique identifier, and transported to the laboratory of parasitology in the Department of Tropical Medicine, University of Kinshasa. Both direct microscopic examination and examination after concentration using the formalin-ether technique (Ritchie technique) were performed [20,21]. The helminthic burden was estimated by counting the total number of eggs of each species observed across all microscopic fields of the preparation, as the intensity of infection could not be expressed in eggs per gram (EPG) due to the technique used [21,22]. Quality control was ensured by re-examining 10% of the samples.

#### 2.4.4. Statistical Analyses

Data were entered into Microsoft Excel and analyzed using EpiInfo 7 and STATA 16.1. Descriptive analyses were used to summarize participant characteristics and estimate the prevalence of geohelminth–malaria co-infection, with 95% confidence intervals. Factors associated with infection were identified using bivariate and multivariate logistic regression models with statistical significance set at *p* < 0.05.

## 3. Results

### 3.1. General Characteristics of Pregnant Women

A total of 336 pregnant women were included in this study. The median age of the participants was 25 years (IQR: 20–31), with nearly half (48.2%) (162/336) being under 25 years of age. The majority were multigravida (72.9%; 245/336) and multipara (42.6%; 143/336). Most participants were in their second trimester of pregnancy (81.6%; 274/336). Nearly 30% (92/336) reported practicing geophagy. The median hemoglobin level was 10.2 g/dL (IQR: 9.2–11.0) with an overall anemia prevalence of 71.7% (241/336). Among anemia cases, 43.4% were moderate and 28.3% mild. The median *P. falciparum* parasite density was 1059 trophozoites/μL of blood (IQR: 270–4057). Additionally, 7.7% participants had at least one geohelminthiasis. Among infected women, the median number of geohelminth eggs observed was 1.5 per slide (IQR: 1–3) (Table 1).

### 3.2. Prevalence of Geohelminth–Malaria Co-Infection in Pregnant Women

The prevalence of geohelminth–malaria co-infection among pregnant women was 5.7% (95% CI: 3.7–8.7) (19/336). The isolated prevalence of geohelminthiasis was 7.7% (95% CI: 5.3–11.1) (26/336), with mono-infections predominating at 7.4% (25/336). Among the identified geohelminths, *A. lumbricoides* was the most common, with a prevalence of 6.3% (95% CI: 4.1–9.4) (21/336), followed by *T. trichiura* at 1.5% (95% CI: 0.64–3.4) (5/336) and *A. duodenale* at 0.3% (95% CI: 0.05–1.7) (1/336) (Table 2).

### 3.3. Factors Associated with Geohelminth–Malaria Co-Infection in Pregnant Women

In bivariate analysis, four factors were associated with geohelminth–malaria co-infection: third trimester of pregnancy (gestational age > 26 weeks), multiparity, multigravidity, and hemoglobin levels above 11 g/dL. However, after adjustment in multivariate analysis, only gestational age > 26 weeks remained significantly associated with co-infection aOR 5.8 (CI 1.59–21.18, *p* = 0.008) (Table 3).

### 3.4. Factors Associated with Geohelminthiasis, Malaria, and Anemia in Pregnant Women

The bivariate analysis also showed an association between geohelminthiasis, multiparity and women with hemoglobin levels ≥ 11 g/dL. Additionally, maternal age (25–30 years; >30 years), multigravidity and multiparity were statistically associated with anemia. However, in the multivariate regression, only gestational age remained statistically linked to geohelminthiasis (Table 4), while multigravidity remained significantly associated with anemia (Table 5).

## 4. Discussion

This study assessed the prevalence of geohelminth–malaria co-infection and identified associated risk factors among pregnant women in Kinshasa. The observed prevalence was 5.7%, with *A. lumbricoides* being the most frequently detected intestinal parasite (6.3%). The third trimester of pregnancy was identified as a significant risk factor for both co-infection and isolated geohelminth infections. However, after adjustment, no significant association was found between co-infection and maternal anemia.

The 5.7% prevalence of geohelminth–malaria co-infection observed in this study is similar to that observed in Ghana (5.7%) [23] and Kenya (6.8%) [11], but lower than the regional average of approximately 20% estimated in a meta-analysis of 24 studies conducted across sub-Saharan Africa [24]. This difference may be attributed to the hospital-based setting and the enhanced monitoring associated with the clinical trial, where participants received preventive interventions such as intermittent preventive treatment (IPTp), use of insecticide-treated nets (ITNs)**,** and systematic deworming during antenatal care visits. Despite these interventions, the persistence of infections highlights the ongoing challenge related to poor sanitation in certain neighborhoods of Kinshasa.

The identification of the third trimester of pregnancy as a significant risk factor for co-infection could be explained by physiological immune alteration occurring in late pregnancy [10]. During this period, cell-mediate immunity is weakened due to an increase in anti-inflammatory cytokines and hormones such as progesterone and cortisol, leading to a reduction in the immune response for T and NK cells. This modulation, which promotes maternal-fetal tolerance, may concurrently increase the susceptibility to parasitic infections such as geohelminthiasis and malaria [10,23,25].

Although nearly one-third of participants reported practicing geophagy, this behavior was not identified as a risk factor in the present study contrary to findings from a previous study indicating that pregnant women practicing geophagy are up to three times more likely to be infected with geohelminths than non-geophagists [6]. This discrepancy may be explained by the enhanced supervision and health education provided to participants as part of a clinical trial, as well as systematic mebendazole administration before stool collection, which could have reduced the parasite load at the time of testing.

The prevalence of isolated geohelminthiasis (7.7%), dominated by *A. lumbricoides* (6.3%), *T. trichiura* (1.5%) and *A. duodenale* (0.3%), may reflect the local environmental characteristics, including high humidity (70–90%), high temperatures (between 25 and 30 °C), and poor waste management and human excreta. Such factors favor the maturation and survival of geohelminth eggs and larvae in the soil [26]. These results are comparable to findings from rural Kenya [27] where 5.6% prevalence was reported among women of childbearing age with hookworms (5.3%) being the predominant helminths, followed by *T. trichiura* (0.6%). However, the prevalence observed here remains lower than that reported in Ethiopia [17], where a study among the general population showed 19.4% prevalence, mainly due to *A. lumbricoides* (14%), *T. trichiura* (4.7%) and hookworms (3.9%).

Approximately 72% of the women presented with mild or moderate anemia, but no significant association was observed between geohelminth–malaria co-infection and maternal anemia after adjustment. This lack of association may be due to regular medical follow-up and systematic supplementation of iron and antiparasitic drugs, which likely mitigated the hematological impact of infection. These findings differ from those of a previous meta-analysis [10] that reported a significantly increased risk of severe anemia in women co-infected with helminth and malaria.

To the best of our knowledge, this is the first hospital-based study in Kinshasa to investigate geohelminth–malaria among pregnant women. Although both infections are endemic in the region, their combined effect during pregnancy women remains poorly documented. Despite the limited number of co-infected cases, our findings provide valuable insight into targeted prevention strategies and improved antenatal care, which could contribute to reducing maternal and neonatal complications. However, the study had some limitations. The relatively low number of co-infected women (19/336) reduced the statistical power of the analyses. Moreover because the participants were enrolled in a clinical trial settings and received standard of care, the results may not fully represent the broader population of pregnant women, many of whom attend antenatal care late or not at all, therefore they do not benefit the available protective measures (iron supplementation, intermittent preventive malaria treatment during pregnancy, distribution of mosquito nets and deworming). To obtain a more accurate estimate of geohelminths prevalence in pregnancy, stool samples should ideally be collected before deworming intervention. Future prospective studies conducted in community settings, incorporating immunological and nutritional markers, are needed to better elucidate the mechanisms underlying geohelminthiasis–malaria co-infection and to more accurately assess its impact on maternal and neonatal health. This research should be conducted outside of the clinical setting in order to understand the true extent of this co-infection within the population.

## 5. Conclusions

Our study revealed a low prevalence of co-infection with geohelminthiasis and malaria among pregnant women. Women in their third trimester of pregnancy were at higher risk of having geohelminthiasis alone and geohelminthiasis–malaria co-infection. Although current interventions (deworming, intermittent preventive malaria treatment) seem to mitigate the impact on anemia, multigravidity emerged as the only risk factor associated with maternal anemia. The persistence of infections underscores the need to strengthen screening among women in their third trimester. Effort should also focus on improving sanitation and hygiene conditions in disadvantaged urban areas, which remain key determinants of geohelminth transmission and adverse maternal outcomes. From a broader perspective, an integrated approach combining parasite control, malaria prevention, and health education is essential to effectively reduce this dual infectious burden, which is particularly concerning among vulnerable pregnant women living in tropical environments.

## Figures and Tables

**Table 1:** Socio-obstetric characteristics of pregnant women enrolled at Lisungi and Lukunga Hospitals, Kinshasa (Democratic Republic of the Congo), November 2022–February 2024.

Variables	N = 336	%	CI (95%)
Age (years)			
<25	162	48.2	42.9–53.6
25–30	84	25	20.7–29.9
>30	90	26.8	22.3–31.8
Median (IQR)	25 (20–31)		
Gestational age (weeks of amenorrhea, WA)			
<26	274	81.6	77.1–85.3
>26	62	18.5	14.7–22.9
Median (IQR)	21.5 (19–25)		
Gestation			
Primigravida	91	27.1	22.6–32.1
Second pregnancy	92	27.4	22.9–32.4
Multigravida	153	45.5	40.3–50.8
Parity			
Nullipara	10	29.7	25.12–34.8
Primipara	93	27.6	23.2–32.7
Multipara	143	42.6	37.4–47.9
Geophagy *			
Yes	92	30.2	25.3–35.5
No	213	69.8	64.5–74.7
Hemoglobin level (g/dL)			
7–9.9	146	43.4	38.3–48.8
10–10.9	95	28.3	23.7–33.3
≥11	95	28.3	23.7–33.3
Median hemoglobin level (IQR)	10.2 (9.2–11)		
Parasite density ° (p/µL)			
<200	43	26.4	19.8–33.8
200–2000	101	61.9	54.0–69.4
>2000	75	34.3	27.9–40.9
Median (IQR)	1059 (270–4057)		
Geohelminths			
Negative	310	92.3	88.9–94.7
Positive	26	7.7	5.3–11.1
Median number of eggs (IQR)	1.5 (1–3)		

° Thick smear: 219 results available for *Pf (P. falciparum)* detection, p/µL: parasite per microliter of blood. * Geophagy: 305 women answered this question. N: sample size IQR: Interquartile range; %: Percentage; CI: Confidence interval; g/dL: grams per deciliter.

**Table 2:** Prevalence and distribution of geohelminthiasis and/or malaria infections in pregnant women at Lisungi and Lukunga hospitals in Kinshasa (Democratic Republic of Congo).

Variables	N	%	CI (95%)
	N = 336		
Geohelminthiasis-malaria co-infection	19	5.7	3.7
Geohelminths	26	7.7	5.3–11.1
Single infection	25	7.4	5.1–10.5
Mixed infection	1	0.3	0.05–1.7
*Ascaris lumbricoides*	21	6.3	4.1–9.4
*Trichuris trichiura*	5	1.5	
*Ancylostoma duodenale*	1	0.3	0.05–1.7
	N = 219		
*Plasmodium falciparum* °	219	100	98.3–100

° Thick smear: 219 results available for Pf *(P. falciparum)* detection.

**Table 3:** Multivariate analysis of factors associated with geohelminthiasis-malaria co-infection among pregnant women at Lisungi and Lukunga Hospitals in Kinshasa (Democratic Republic of the Congo), November 2022–February 2024.

Variables	Geohelminthiasis- Malaria		cOR	CI (95%)	*p*-Value	aOR	CI (95%)	*p*-Value
	N	Co-Infected						
Age (years)								
<25	162	13	1			1		
25–30	84	3	0.4245	0.12–1.53	0.191	1.0547	0.14–7.79	0.958
>30	90	3	0.3952	0.11–1.43	0.156	4.9477	0.46–53.4	0.188
Gestational age (weeks of amenorrhea)								
<26	274	11	1			1		
>26	62	18.5	3.5420	1.36–9.22	0.010	5.8100	1.59–21.2	0.008
Gravida								
Primigravida	91	15	1			1		
Second pregnancy	92	3	0.2451	0.07–0.91	0.036	0.0940	0.003–2.87	0.175
Multigravida	153	1	0.2457	0.08–0.73	0.012	0.2565	0.007–9.96	0.466
Parity								
Nullipara	10	11	1			1		
Primipara	93	5	0.4597	0.15–1.38	0.165	2.2218	0.07–67.7	0.647
Multipara	143	3	0.1733	0.05–0.64	0.008	0.1428	0.004–5.0	0.284
Geophagy *								
No	213	10	1			1		
Yes	92	8	1.9333	0.74–5.07	0.180	1.8737	0.61–5.79	0.275
Hemoglobin level (g/dL)								
7–9.9	146	15	1			1		
10–10.9	95	3	0.2847	0.08–1.01	0.052	0.3242	0.06–1.68	0.179
≥11	95	1	0.0929	0.01–0.72	0.023	0.2124	0.02–1.87	0.163
Parasite density ° (p/µL)								
<200	43	4	1			1		
200–2000	101	8	0.8387	0.24–2.95	0.784	0.4289	0.09–2.02	0.284
>2000	75	7	1.0037	0.28–3.65	0.996	0.8883	0.19–4.13	0.880

° Thick smear: 219 results available for Pf (*P. falciparum*) detection; p/µL: parasite per microliter of blood. * Geophagy: 305 women answered this question. N (sample size); cOR: crude odds ratio; aOR: adjusted odds ratio; %: percentage; CI: confidence interval; g/dL: grams per deciliter.

**Table 4:** Multivariate analysis of factors associated with geohelminthiasis among pregnant women at Lisungi and Lukunga Hospitals in Kinshasa (Democratic Republic of the Congo), November 2022–February 2024.

Variables	Geohelminthiasis		cOR	CI (95%)	*p*-Value	aOR	CI (95%)	*p*-Value
	N	Parasitized						
Age (in years)								
<25	162	17	1			1		
25–30	84	4	0.426	0.13–1.31	0.137	1.054	0.14–7.79	0.958
>30	90	5	0.501	0.17–1.40	0.190	0.497	0.45–53.44	0.188
Gestational age (in weeks)								
<26	274	18	1			1		
>26	62	8	2.106	0.87–5.09	0.098	5.810	1.59–21.18	0.008
Gestation								
Primigravida	91	11	1			1		
Second pregnancy	92	8	0.692	0.26–1.81	0.454	0.094	0.003–2.87	0.175
Multigravida	153	7	0.348	0.13–0.93	0.036	0.256	0.007–9.95	0.466
Parity								
Nullipara	100	11	1			1		
Primipara	93	10	0.974	0.39–2.41	0.956	2.221	0.07–67.73	0.647
Multipara	143	5	0.293	0.09–0.87	0.027	0.142	0.004–5.03	0.284
Geophagy *								
No	213	15	1			1		
Yes	92	11	1.609	0.69–3.73	0.267	1.873	0.60–5.79	0.275
Hemoglobin level (g/dL)								
7–9.9	146	17	1			1		
10–10.9	95	6	0.511	0.19–1.34	0.175	0.324	0.62–1.67	0.179
≥11	95	3	0.247	0.07–0.86	0.029	0.212	0.0–1.86	0.163
Parasite density ° (p/µL)								
<200	43	4	1			1		
200–2000	101	8	0.838	0.23–2.94	0.784	0.428	0.09–2.01	0.284
>2000	75	7	1.003	0.27–3.64	0.996	0.888	0.19–4.13	0.880

° Thick smear: 219 results available for Pf *(P. falciparum)* detection; p/µL: parasite per microliter of blood. * Geophagy: 305 women answered this question. N (sample size) cOR: crude odds ratio; aOR: adjusted odds ratio; %: percentage; CI: confidence interval; g/dL: grams per deciliter.

**Table 5:** Multivariate analysis of factors associated with maternal anemia among pregnant women at Lisungi and Lukunga Hospitals in Kinshasa (Democratic Republic of the Congo), November 2022–February 2024.

Variables	Anemia (Hb ≤ 11 g/dL)		cOR	CI (95%)	*p*-Value	aOR	CI (95%)	*p*-Value
	N	Yes						
Age (in years)								
<25	162	127	1			1		
25–30	84	57	1.913	1.06–3.43	0.030	1.506	0.53–4.27	0.441
>30	90	59	1.906	1.07–3.38	0.027	0.523	0.16–1.70	0.283
Gestational age (in weeks)								
<26	274	193	1			1		
>26	62	48	0.694	0.36–1.33	0.272	0.383	0.13–1.09	0.073
Gestation								
Primigravida	91	76	1			1		
Second pregnancy	92	68	1.788	0.86–3.68	0.115	2.047	0.70–5.96	0.189
Multigravida	153	97	2.925	1.53–5.57	0.001	3.678	1.08–12.5	0.037
Parity								
Nullipara	100	81	1			1		
Primipara	93	72	1.243	0.61–2.49	0.540	0.225	0.12–1.21	0.103
Multipara	143	88	2.664	1.45–4.86	0.001	1.474	0.16–1.37	0.170
Geophagy *								
No	213	156	1			1		
Yes	92	62	1.324	0.77–2.25	0.300	1.355	0.60–3.06	0.465
Geohelminths								
No	310	218	1			1		
Yes	26	23	0.309	0.09–1.05	0.061	0.266	0.03–2.16	0.216
Parasite density ° (p/µL)								
<200	43	28	1			1		
200–2000	101	78	0.550	0.25–1.20	0.134	0.450	0.17–1.14	0.094
>2000	75	61	0.428	0.18–1.001	0.052	0.389	0.13–1.08	0.071

° Thick smear: 219 results available for *Pf (P. falciparum)* detection * Geophagy: 305 women answered this question. N: sample size cOR: crude odds ratio; aOR: adjusted odds ratio; %: percentage; CI: confidence interval.

## Data Availability

The datasets generated and/or analyzed during the current study are available from the corresponding author on reasonable request.

## Abbreviations

ANC	Antenatal care
CI	Confidence interval
DRC	Democratic Republic of the Congo
EDCTP	European & Developing Countries Clinical Trials Partnership
Hb	Hemoglobin
IQR	Interquartile range
ORa	Adjusted odds ratio
ORc	Crude odds ratio
PACTR	Pan African Clinical Trials Registry
Pf	*Plasmodium falciparum*
